# The Genetic Diversification of a Single Bluetongue Virus Strain Using an In Vitro Model of Alternating-Host Transmission

**DOI:** 10.3390/v12091038

**Published:** 2020-09-18

**Authors:** Jennifer H. Kopanke, Justin S. Lee, Mark D. Stenglein, Christie E. Mayo

**Affiliations:** Department of Microbiology, Immunology, and Pathology, Colorado State University, Fort Collins, CO 80523, USA; jennifer.kopanke@wsu.edu (J.H.K.); justin.lee@colostate.edu (J.S.L.); mark.stenglein@colostate.edu (M.D.S.)

**Keywords:** bluetongue virus, *Orbivirus*, viral evolution, viral populations, invertebrate host, vertebrate host, selection

## Abstract

Bluetongue virus (BTV) is an arbovirus that has been associated with dramatic epizootics in both wild and domestic ruminants in recent decades. As a segmented, double-stranded RNA virus, BTV can evolve via several mechanisms due to its genomic structure. However, the effect of BTV’s alternating-host transmission cycle on the virus’s genetic diversification remains poorly understood. Whole genome sequencing approaches offer a platform for investigating the effect of host-alternation across all ten segments of BTV’s genome. To understand the role of alternating hosts in BTV’s genetic diversification, a field isolate was passaged under three different conditions: (i) serial passages in *Culicoides sonorensis* cells, (ii) serial passages in bovine pulmonary artery endothelial cells, or (iii) alternating passages between insect and bovine cells. Aliquots of virus were sequenced, and single nucleotide variants were identified. Measures of viral population genetics were used to quantify the genetic diversification that occurred. Two consensus variants in segments 5 and 10 occurred in virus from all three conditions. While variants arose across all passages, measures of genetic diversity remained largely similar across cell culture conditions. Despite passage in a relaxed in vitro system, we found that this BTV isolate exhibited genetic stability across passages and conditions. Our findings underscore the valuable role that whole genome sequencing may play in improving understanding of viral evolution and highlight the genetic stability of BTV.

## 1. Introduction

Bluetongue virus (BTV; family *Reoviridae*, genus *Orbivirus*) is a globally distributed, arthropod-borne virus that can cause profound disease in both domestic and wild ruminants. BTV is the etiologic agent of bluetongue disease and is transmitted by biting midges in the genus Culicoides. Bluetongue is listed as a notifiable disease by the World Organisation for Animal Health (OIE). Clinical signs in infected animals may vary; some animals may not exhibit any clinical signs, while others may develop fever, mucosal ulcerations, pneumonia, or coronitis [1,2]. Sheep and white-tailed deer are considered to be relatively more susceptible to severe or even fatal disease compared to other ruminants, although cattle and other wildlife species are also vulnerable to infection and illness.

The global distribution of bluetongue virus is defined by the presence of a competent insect vector, which is—with few exceptions—necessary for virus transmission between ruminants [3,4]. In North America, the predominant vector species is *Culicoides sonorensis*, although additional vectors exist in certain locations such as Florida (i.e., *C. insignis*) [5,6,7,8]. *Culicoides* midges that become infected with BTV through an infective blood meal do not appear to manifest adverse effects and remain persistently infected and capable of transmitting virus to ruminant hosts throughout their life [9].

The BTV genome is composed of ten segments of double-stranded RNA (dsRNA) which encode 12 distinct proteins that make up the viral particle and its machinery. Given its segmented, dsRNA composition, BTV can evolve via several mechanisms, including through the occurrence of mutation and reassortment in a viral population.

Whole genome sequencing of BTV field isolates indicates that reassortment may occur frequently among BTV strains, and in vivo and in vitro studies have similarly shown that certain strains of BTV can reassort readily [10,11,12,13,14]. However, less is known regarding the role that mutation plays in BTV’s genetic diversification.

High rates of mutation and large viral population sizes appear to contribute to the overall ability of many arboviruses to maintain fitness in vertebrate and invertebrate hosts, but it is not known whether similar factors come into play for BTV during its transmission cycle [15,16,17]. Arboviruses generally experience purifying selection and evolve more slowly than conventionally transmitted viruses, reportedly due to the selective pressures exerted by transmission between highly divergent hosts [16,18,19,20,21,22,23,24,25,26]. Moreover, studies of BTV and other dsRNA viruses indicate that dsRNA viruses tend to have lower substitution rates over time, perhaps in part due to their double-stranded genome composition [24,27]. 

While it is presumed that purifying selection plays an essential role in the maintenance of viral fitness in BTV transmission, only a handful of studies have investigated BTV’s genetic evolution in vertebrate and invertebrate hosts [20,28,29,30]. These studies predominantly occurred before the advent of next-generation, whole-genome sequencing (WGS), and as a result only were able to characterize the genetic changes occurring within one or two segments of the BTV genome, or were based upon earlier methods to detect genetic differences, such as electropherotype [20,28,29,30]. It remains unclear whether each segment experiences similar selection pressures across transmission cycles, or whether there are differential effects across the ten genome segments. Moreover, insect and mammalian hosts presumably exert differing selection pressures that have the potential to be amplified when alternating transmission is eliminated.

The aim of this study, therefore, was to determine the genetic stability of BTV across its entire genome when passaged in a relaxed in vitro system emulating BTV’s natural transmission cycle. Although there is good evidence that at least two segments remain relatively unchanged through 2–3 alternating passages in *Culicoides sonorensis* and domestic ruminants, to date there has not been a robust investigation of the effect of BTV’s alternating life cycle across all ten genome segments over multiple generations [20]. Here, we use an in vitro system that leverages cell lines derived from two of BTV’s natural hosts (*Culicoides sonorensis* and cattle) and whole genome sequencing to answer fundamental questions regarding the population makeup and genetic diversity of this virus as it alternates between host systems.

## 2. Materials and Methods

### 2.1. Virus Isolation

A field isolate of BTV-17 from California (GenBank, MT952971 – MT952980) was isolated from BTV-positive whole blood during a naturally occurring infection in a clinically affected sheep and passaged as previously described by DeMaula et al. [31].The virus was expanded once prior to initiation of the current experiment by a single passage in BHK 21 cells and infectious titer was determined via 50% tissue culture infectious dose (TCID50) using the Reed–Muench method [32].

### 2.2. Cells

Bovine pulmonary artery endothelial cells (BPAEC) were maintained in Advanced MEM (Gibco, Dublin, Ireland) supplemented with 1% non-essential amino acids, 1% penicillin-streptomycin (10,000 U/mL), and 10% heat-inactivated fetal bovine serum (FBS). Cells were held at 37 °C with 5% CO_2_ supplementation and were passaged every 3–4 days when approximately 80–90% confluent.

CuVaW3 cells, derived from *Culicoides sonorensis* embryos, were maintained in a modified Schneider’s Drosophila Media supplemented with 15% FBS and passaged every 3–4 days when ~90% confluent (File S1) [33,34]. CuVaW3 cells were held at 27 °C without additional CO_2_ supplementation. 

### 2.3. Virus Infections

BTV-17 (BTV17-INPUT) was used to infect confluent monolayers of BPAEC or CuVaW3 cells at an MOI of 1 in duplicate under three different conditions. Virus was either passaged serially in BPAEC (BTV17-BPAEC), serially in CuVaW3 cells (BTV17-CUVA), or alternatingly between bovine and insect cell lines (BTV17-ALT) for 10 consecutive passages.

To establish virus infections, BTV was diluted in EMEM (for BPAEC infections) or modified Schneider’s Drosophila Media (for CuVaW3 infections) to reach an MOI of 1 TCID50/mL. One ml of diluted virus was then added to confluent monolayers of BPAEC or CuVaW3 cells in 25 cm^2^ flasks. Additional flasks of each cell type were inoculated with EMEM or modified Schneider’s Drosophila Media only as negative controls. After 1 h of incubation at 37 °C or 27 °C depending on cell type, an additional 4 mL of maintenance media was added to each flask. Inoculated cultures were maintained at 37 °C with 5% CO_2_ supplementation (BPAEC) or at 27 °C without additional CO_2_ supplementation (CuVaW3) until harvest. After initial infection, virus was passaged blindly every ~96 h to avoid freeze–thaw cycles.

Virus was harvested from each passage when bovine cells showed >80 % cytopathic effect (CPE). Insect cells did not demonstrate CPE, but the presence of BTV was confirmed with RT-PCR at each passage. Virus collected from each passage was used to initiate each subsequent round of infection, and remaining stocks were stored immediately at −80 °C for downstream applications. At each passage, the same volume of virus supernatant from each respective lineage was used to initiate the subsequent round of infection. In this way, we attempted to maintain an equal MOI in each condition, while avoiding free–thaw cycles.

### 2.4. RT-PCR

Nucleic acid from viral supernatant collected at each passage was extracted using a MagMAX Pathogen RNA/DNA kit (Applied Biosystems, Foster City, CA, USA) according to the manufacturer’s instructions for low cell content samples. Extracted samples were prepared for RT-PCR using a universal one-tube fluorogenic probe-based reaction that detects BTV segment 10 as described by Hoffman et al. and modified as outlined by Ortega et al. [35,36]. Reactions were prepared using a SuperScript^TM^ III One-step RT-PCR kit (Invitrogen, Carlsbad, CA, USA) at half-reaction volumes and were thermocycled as previously described [35].

### 2.5. WGS Library Preparation

Input virus (BTV17-INPUT) and duplicates from each condition (BTV17-CUVA, BTV17-ALT, and BTV17-BPAEC) collected after passages 1, 3, 6, 9, and 10 were prepared for whole genome sequencing (WGS). Extracted samples were treated with 4 U of DNase (TURBO DNA-*free*^TM^ kit, Invitrogen) according to manufacturer’s instructions. DNased nucleic acids were then incubated with LiCl (final concentration 2.0 M) for 14–18 h at 4 °C to selectively precipitate single stranded RNA and maximize dsRNA yield. Following incubation, samples were centrifuged at 4 °C × 20 min at 18,000× *g*. Supernatant was collected and excess salts were removed via a 1.25× MagMAX Pathogen RNA/DNA kit clean-up step.

Libraries for each sample were then prepared for whole genome metagenomic sequencing using a ScriptSeq v2 RNA-Seq library preparation kit (Epicentre, Madison, WI, USA) according to the manufacturer’s instructions, except RNA fragmentation time was reduced to 2 min 30 s at 85 °C. Adapters containing unique 6-mer barcodes (ScriptSeq Index PCR Primers, Epicentre) were annealed to each sample. Libraries were cleaned using a 1x Agencourt AMPure XP (Beckman Coulter, Brea, CA, USA) magnetic bead-based clean-up, and concentration and quality of each library was measured using Agilent’s High Sensitivity D1000 ScreenTape assay on the TapeStation 2200 instrument (Agilent, Santa Clara, CA, USA). Samples were pooled to achieve roughly equal concentrations prior to size-selection. Pooled, indexed products between 300–700 base pairs (bp) in length were manually selected by fractionating the pooled library on a 1% agarose gel, followed by excising the desired region and performing gel extraction according to kit instructions (QIAquick Gel Extraction Kit, Qiagen, Hilden, Germany). Concentration, quality, and size-distribution of pooled, size-selected libraries were then once again determined via High Sensitivity D1000 ScreenTape. Library concentration was quantified using the KAPA Library Quantification qPCR kit (KAPA Biosystems, Basel, Switzerland) according to manufacturer’s instructions.

Four initial samples were sequenced on the Illumina MiSeq instrument using 300 cycle (2 × 150) MiSeq v2 reagents (Illumina Inc., San Diego, CA, USA). Subsequently, batches of 15–16 samples were sequenced on the Illumina NextSeq mid-output 300 cycle (2 × 150) v2 reagents (Illumina Inc.) to achieve sufficient sequencing depth across all ten segments.

### 2.6. Bioinformatics

Libraries were demultiplexed and reads from each sample were quality-filtered via a pre-processing bioinformatics pipeline that uses trimmomatic to remove bases and sequences with low quality scores, as well as adapter sequences [37]. Trimmed reads were then processed using Cd-hit to eliminate duplicate reads (those where two or more reads had ≥ 96% pairwise identity in the first and last 30 base pairs) [38]. Reads were then aligned to the consensus sequence of the parental input virus (BTV17-INPUT) in Bowtie2 using default parameters [39]. Finalized sequences were examined in Geneious v.10.2.2 to confirm alignment accuracy.

Quality-filtered reads in BAM format from BTV17-INPUT and virus replicates from passages 3, 6, and 9 were analyzed for single nucleotide variants (SNVs) and insertions–deletions (indels) using LoFreq [40]. Indel qualities were added to BAM data using a --lofreq indelqual with --dindel option. Default LoFreq parameters, which include stringent thresholds based on alignment quality, base quality, and mapping quality, were used for SNV and indel detection. Only LoFreq-detected variants in the coding sequence of each segment were included in downstream analyses.

Variants with significant strand-bias were pre-filtered based on LoFreq default parameters. Output .vcf files were imported into Geneious v.10.2.2 and visually inspected along with alignments.

### 2.7. Population Genetics

Viral population diversity was assessed in several ways. Genetic distance was determined for each sample by summing coding sequence SNV frequencies for each segment [41]. Richness was also measured using viral population-specific modifications: the number of SNV sites detected within the coding sequence of each of the ten genome segments was tabulated for each sample, and then normalized by the total number of BTV reads aligning per segment [41,42]. Richness was calculated as the number of unique SNV sites (i.e., unique nucleotide positions where one or more alternate alleles may be present) present per 10,000 BTV reads.

Shannon entropy across samples and segments was calculated as a measure of population complexity to better characterize the genetic makeup of the viral milieu generated in each condition. The following equation, based on previously published work, was used:

$$S_{i,s}=-p_{s}\left(\ln p_{s}\right)+\left(1-p_{s}\right)\times \ln\left(1-p_{s}\right)$$

where the within-host viral population’s Shannon entropy ($S_{i,s}$) is estimated as the mean *S* across all nucleotide positions (*s*) using the SNV frequency (*p*) at each nucleotide position [41]. Mean Shannon entropy across all sites was determined for each segment and/or sample.

Changes in the fixation index ($F_{ST}$) between the input virus and along the lineage of each replicate were calculated as a measure of genetic divergence across conditions and passages. $F_{ST}$ was estimated by the method-of-moments technique as described by Reynolds and refined for WGS datasets by Fumagalli et al. [43,44]. As has been performed for other viral deep sequencing datasets, the number of individuals sampled in each population (*n*), was set to the mean BTV coverage depth for each segment’s coding sequence (segment 1: 1604; segment 2: 2423; segment 3: 1375; segment 4: 1726; segment 5: 3014; segment 6: 3217; segment 7: 1866; segment 8: 5332; segment 9: 5282; segment 10: 2664) [41]. The frequencies of non-reference variants (those that differed from the consensus sequence) were estimated as $p_{i,\;s}$, $p_{j,s}$ and $p_{s}$_,_ for populations *i*, *j*, and *i+j*, respectively, at site *s*. All other sites where non-reference variants were not detected were set to *p* = 0. Genetic variance at a single site, *s*, was then calculated based on the following equations:

$$a_{s}=\frac{\left(4n_{i}{\left(p_{i,s}-p_{s}\right)}^{2}+4n_{j}{\left(p_{j,s}-p_{s}\right)}^{2}-b_{s}\right)}{2(2n_{i}n_{j}/\left(n_{i}+n_{j}\right)}$$

and

$$b_{s}=\frac{\left(n_{i}\alpha_{i,s}+n_{j}\alpha_{j,s}\right)}{\left(n_{i}+n_{j}-1\right)}$$

where

$$\alpha_{i,s}=2p_{i,s}\left(1-p_{i,s}\right),\;\mathrm{and}\;\alpha_{j,s}=2p_{j,s}\left(1-p_{j,s}\right)$$

$F_{ST}$ at a single site *s* was then estimated as $\frac{a_{s}}{\left(a_{s}\;+\;b_{s}\right)}$, and cumulatively across a coding sequence locus (*m* sites) as $\frac{\sum_{s=1}^{m}a}{\sum_{s=1}^{m}\left(a+b\right)}.$ $F_{ST}$ between input and passaged viruses and along lineages of each replicate was calculated for each segment. $F_{ST}$ between two populations may range from 0 to 1, with an $F_{ST}$ of 1 representing highly divergent populations. This measure was applied to understand how viral populations shifted from BTV17-INPUT (passage 0) to passage 3, and then within each replicate, how populations diverged from passages 3 to 6 and 6 to 9.

To estimate degree of selection, $d_{N}/d_{S}$ was calculated for each sample. Total nonsynonymous (*N_s_*) and synonymous (*S_s_*) sites for the coding region of each segment across passages 3, 6, and 9 were determined using DnaSP 6 via the Nei–Gojobori method [45,46]. Based on recommendations for viral data, nonsynonymous substitutions (*N_d_*) and synonymous substitutions (*S_d_*) were calculated as the sum of nonsynonymous and synonymous substitution frequencies, respectively [41,47]. The $d_{N}/d_{S}$ ratio was then calculated based on the Jukes–Cantor formula [48]:

$$d_{S}\;=\;\frac{-3\;\times \;\ln\left(1-\left(\left(4S_{d}/S_{s}\right)/3\right)\right)}{4}$$

and

$$d_{N}\;=\;\frac{-3\;\times \;\ln\left(1-\left(\left(4N_{d}/N_{s}\right)/3\right)\right)}{4}$$

A $d_{N}/d_{S}$ ratio > 1 suggests positive selection, while a $d_{N}/d_{S}$ ratio of <1 indicates negative selection. While $d_{N}/d_{S}$ provides an estimate of selection, it is considered a relatively insensitive measure for intra-host virus populations, and is therefore only interpreted as a guide towards general trends in this dataset [49].

### 2.8. Statistics

Statistical analyses were carried out using GraphPad Prism 8.1.0. Unless otherwise noted, two-way repeated measures ANOVA with Tukey’s post-hoc test was used to analyze the effect of condition (CuVaW3, BPAEC, or alternating propagation) and genome segment on measures of viral population genetics, with a *p* < 0.05 considered significant.

## 3. Results

### 3.1. Whole Genome Sequencing and Detection of Single Nucleotide Consensus Changes

**Figure 1:**
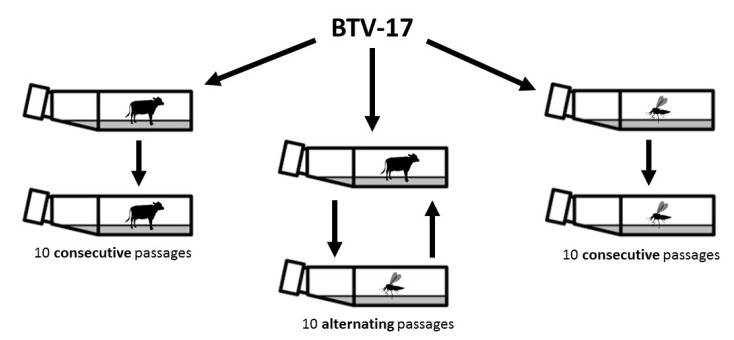
Experimental Set-up. A field isolate of bluetongue virus (BTV)-17 (BTV17-INPUT) was passaged under three different cell culture conditions: serial passages in bovine cells (BTV17-BPAEC); serial passages in *Culicoides sonorensis* cells (BTV17-CUVA); and alternating passages in bovine and *C. sonorensis* cells (BTV17-ALT) for 10 consecutive passages.

Molecular techniques were used to assess the impact of alternating host transmission on BTV’s genetic diversification over time (Figure 1). RT-PCR was used as an estimate of viral replication. Ct values remained consistently low across conditions and passages (mean: 14.6; standard deviation (SD): 0.7; range: 13.4–16). We attempted to determine infectious titer of passaged viruses using TCID50 assays on BHK21 cells, but the cytopathic effect was not reliably detectable even at the lowest dilutions (10^−1^) in our standard 96 h assay. We suspect that samples may have been compromised during storage, or that BTV passaged serially on BPAEC or CuVaW3 cells may have diminished growth kinetics in BHK 21 cells, thereby compromising our ability to accurately titer this virus using our standard assay parameters. As a result, we used BTV Ct values as a measure of viral replication across passages.

Whole genome sequencing was coupled with variant detection to establish single nucleotide variant (SNV) frequencies for the input (BTV17-INPUT) and passaged viruses. Depth of BTV coverage across the coding sequences of all samples and segments varied (Table S1), with a mean depth of 3529 (SD: 2066). Only coding sequence SNVs above 0.2% frequency and without significant strand bias (as identified by LoFreq default parameters) and depth > 100 reads were included in analyses. The mean depth at positions with variants across all samples was 3084 (SD: 2541).

While the occurrence of SNVs varied across samples, the consensus sequence of BTV17-INPUT shared 100% nucleotide identity with output viruses across all conditions in segments 1, 2, 3, 4, 6, 7, 8, and 9, and >99.8% nucleotide identity in segments 5 and 10. Consensus sequences for BTV17-CUVA, BTV17-ALT, and BTV17-BPAEC were identical across all ten segments at each time point (passages 1, 3, 6, 9, and 10).

Single nucleotide consensus changes (i.e., variants with >50% frequency) arose and approached fixation in segments 5 (nonsynonymous, residue 229I → R) and 10 (synonymous, nucleotide 360A → G) after a single passage in CuVaW3 cells or BPAEC cells. These changes were conserved across remaining passages in all three cell culture conditions (segment 5 229I → R frequency: 99.35–99.96%, and segment 10 360A → G frequency: 97.39–99.89% across all samples). No further consensus changes occurred with additional passages.

Resequencing of an additional aliquot of the original BTV17-INPUT virus confirmed that neither variant was present as the consensus nucleotide in the input virus. The segment 5 229I → R variant was detected in 0 of 685 reads at that site in the initially sequenced input virus, and in only 1 of 1175 reads at that site (0.05% frequency) in the subsequent aliquot. In contrast, the segment 10 360A → G variant was detected at approximately 20% frequency in both aliquots of the input virus.

### 3.2. Measures of Genetic Diversity across Passages and Cell Types

**Figure 2:**
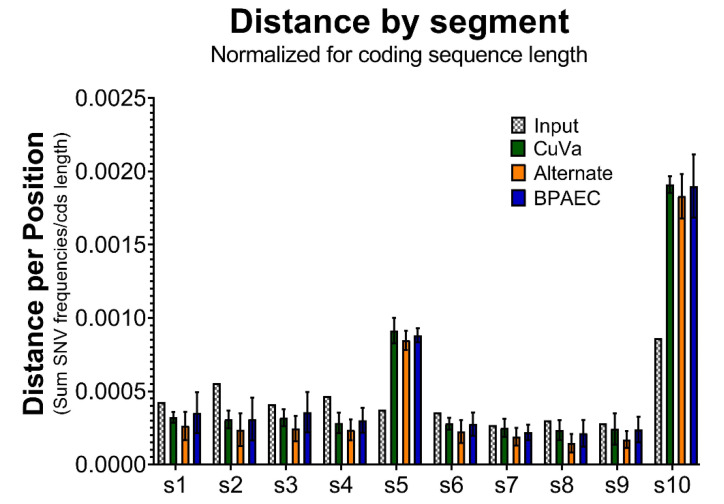
BTV Genetic Distance by Segment across Cell Culture Conditions. Genetic distances (the sum of single nucleotide variant (SNV) frequencies per segment) were normalized by each segments’ coding sequence length. Segments 1–10 are represented along the *x*-axis (s1, s2, s3, s4, s5, s6, s7, s8, s9, and s10). For BTV17-CUVA, -ALT, and -BPAEC, mean distance (and standard deviation) for each segment across passages 3, 6, and 9 is shown.

Whole genome sequencing data were analyzed for low frequency variants and insertions–deletions (indels) and various measures of genetic diversity, were assessed to better understand genetic variation in a relaxed, in vitro model of BTV transmission. Genetic distance was approximated as the sum of all SNV frequencies per coding sequence. BTV17-CUVA, BTV17-ALT, and BTV17-BPAEC exhibited similar genetic distances. Consistent with the consensus mutations that occurred in passaged viruses, BTV17-CUVA, BTV17-ALT, and BTV17-BPAEC demonstrated nearly two-fold greater genetic distance compared to BTV17-INPUT in segments 5 and 10 (Figure 2). When assessed for trends during the progression of passages, viruses exhibited similar genetic distances across all 10 segments regardless of cell culture condition (Figure S1).

**Figure 3:**
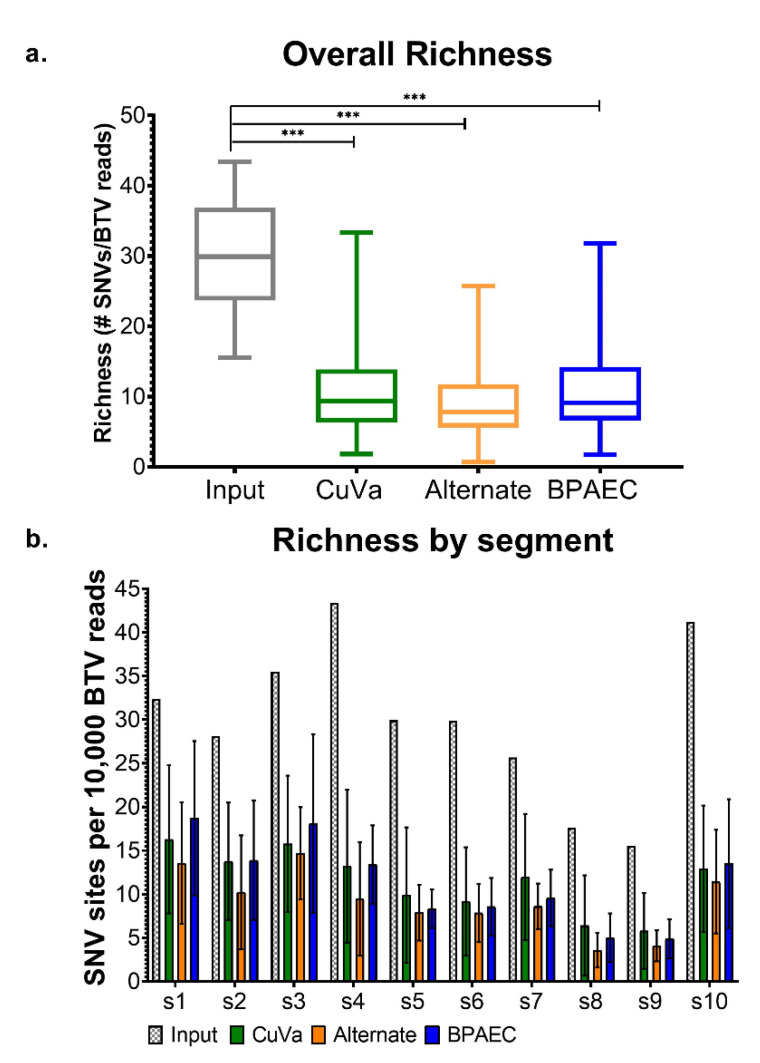
BTV Genetic Richness across Cell Culture Conditions. (**a**) Richness of each segment was calculated as the sum of single nucleotide variants (SNV) sites normalized by the number of BTV reads (i.e., variant sites per 10,000 BTV reads), and collective data across all segments are shown by box-and-whisker plots (median, interquartile range, and minimum/maximum are depicted; *** = *p* < 0.005). Box-and-whisker plots for BTV17-CUVA, -ALT, and -BPAEC were constructed using the richness of all segments across passages 3, 6, and 9. (**b**) Mean richness and standard deviation for each segment are shown. Segments 1–10 are represented along the *x*-axis (s1, s2, s3, s4, s5, s6, s7, s8, s9, and s10). Bars depicting BTV17-CUVA, -ALT, and -BPAEC represent collective data from passages 3, 6, and 9.

There was relatively wide variation in richness across samples and segments (Figure 3). When analyzed across all three cell culture conditions (BTV17-CUVA, -ALT, and -BPAEC), overall richness was significantly lower in segments 8 and 9 than in the other eight segments (*p* < 0.0001). Segments 1 and 3 demonstrated the highest overall richness (*p* < 0.05). Interestingly, richness across the entire coding sequence of BTV17-INPUT was greater than that detected in any of the subsequent passages, regardless of cell culture condition (*p* < 0.0005) (Figure 3a). BTV17-CUVA, BTV17-ALT, and BTV17-BPAEC demonstrated substantial variability in richness within each segment across passages (Figure S2).

**Figure 4:**
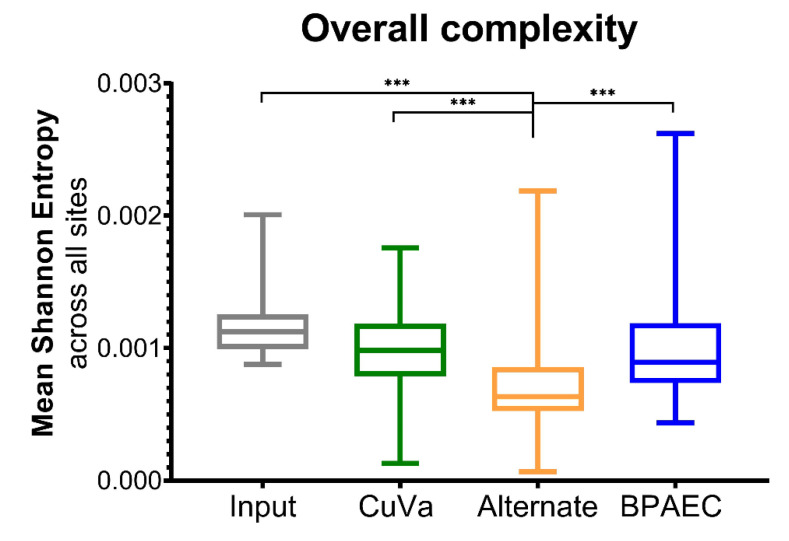
BTV Genetic Complexity across Cell Culture Conditions. Shannon entropy was calculated as a measure of population complexity across viral coding sequences. Shannon entropy was calculated for each segment and cumulative data from all segments are shown by box-and-whisker plots (median, interquartile range, and minimum/maximum are depicted; *** = *p* < 0.005). Box-and-whisker plots for BTV17-CUVA, -ALT, and -BPAEC were constructed using the mean Shannon entropy of all segments across passages 3, 6, and 9.

**Figure 5:**
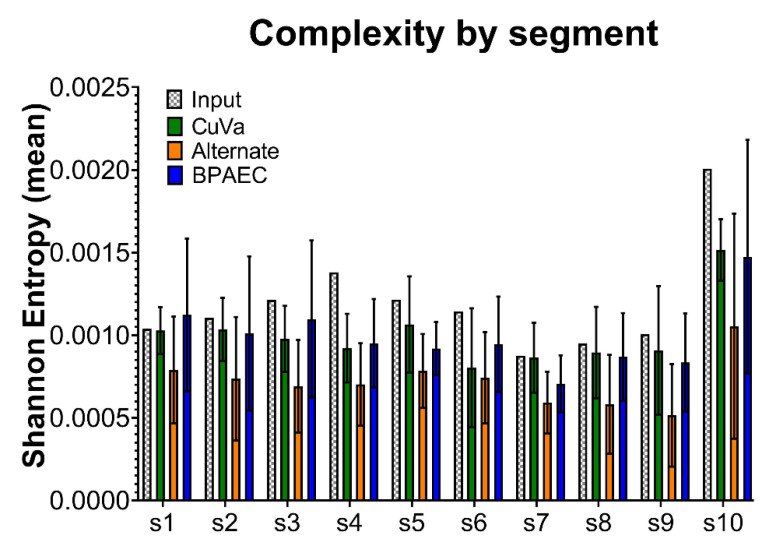
BTV Genetic Complexity by Segment across Cell Culture Conditions. Shannon entropy was calculated for each segment as a measure of viral population complexity. Segments 1–10 are represented along the *x*-axis (s1, s2, s3, s4, s5, s6, s7, s8, s9, and s10). For BTV17-CUVA, -ALT, and -BPAEC, mean Shannon entropy (and standard deviation) for each segment across passages 3, 6, and 9 are shown.

We then measured population complexity using Shannon entropy as an estimator of uncertainty within a viral population. Cumulatively across passages, BTV17-ALT was the least complex viral population, with significantly lower Shannon entropy than BTV17-INPUT, BTV17-CUVA, and BTV17-BPAEC (*p* < 0.005) (Figure 4). When analyzed for segment-specific trends, segment 10 demonstrated the greatest Shannon entropy across all conditions (*p* < 0.05) (Figure 5). Segment-specific trends were not detected between cell culture conditions across passages (Figure S3).

**Figure 6:**
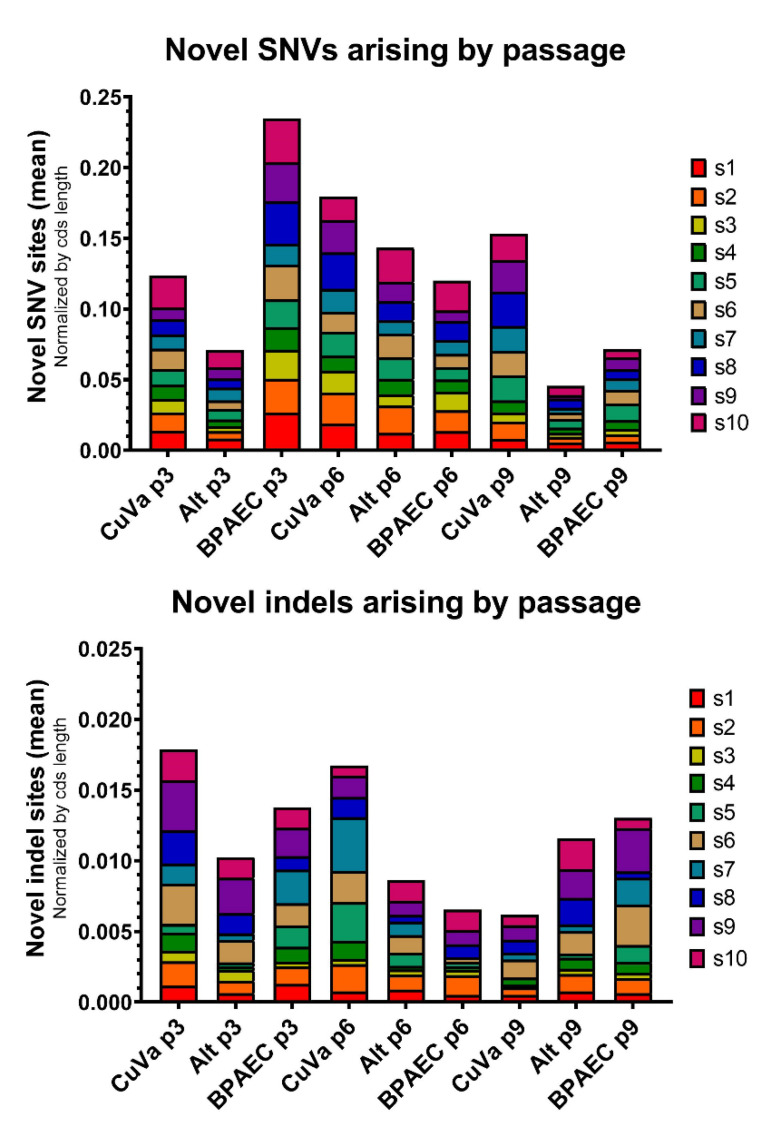
SNVs and Indels by Passage and Cell Culture Condition. The total number of novel SNVs or indels was calculated for each sample and normalized by the nucleotide length of the coding sequence (CDS) of each segment. The mean number of normalized novel sites per segment is plotted according to passage and cell culture condition. Segments 1–10 are represented in each bar graph (s1, s2, s3, s4, s5, s6, a7, s8, s9, s10). Viruses harvested from each condition at passage 3 (CuVa p3, Alternate (Alt) p3, and BPAEC p3), passage 6 (CuVa p6, Alt p6, BPAEC p6) and passage 9 (CuVa p9, Alt p9, BPAEC p9) are depicted.

Novel SNVs and indels arose in each condition and passage, although the number of new variants varied across samples (Figure 6). The occurrence of novel indels—which are presumed to be universally deleterious when associated with shifts in reading frame—was approximately ten-fold less than the occurrence of novel SNVs. Several novel variants and indels re-occurred across samples or passages, disappearing in one passage and then reoccurring later (data available upon request). This may reflect a predisposition for variants or indels to occur repeatedly in certain parts of the genome, or it may indicate subtle variations in sequencing quality that resulted in failure to detect these low-level variants in certain samples.

### 3.3. Measures of Genetic Divergence across Passages and Cell Types

Genetic divergence was estimated by calculation of the fixation index ($F_{ST})$ to understand how viral populations shifted across passages. Marked divergence from BTV17-INPUT was detected in segments 5 and 10 across all samples (segment 5 mean $F_{ST}$ = 0.67 (range 0.64–0.70); segment 10 mean $F_{ST}$ = 0.73 (range 0.70–0.74)), consistent with consensus changes that arose in these segments. Segments 1 and 2 also demonstrated modest divergence from BTV17-INPUT (mean $F_{ST}$ 0.12 and 0.24, respectively) across all samples by passage 3. The remaining segments had very low $F_{ST}$ values from BTV17-INPUT (passage 0) to passage 3 viruses, with segments 6, 8, and 9 demonstrating the lowest divergence from the input virus across all three conditions. Subsequent to initial passages, when most marked divergence was detected, $F_{ST}$ values exhibited relatively consistent rates of divergence between passages 3 and 6, and passages 6 and 9. $F_{ST}$ values were similar among cell culture conditions and segments, and while BTV17-ALT showed slightly higher $F_{ST}$ values than matched BTV17-CUVA and BTV17-BPAEC samples across all segments, this trend was non-significant.

### 3.4. Measures of Selection across Passages and Cell Types

**Figure 7:**
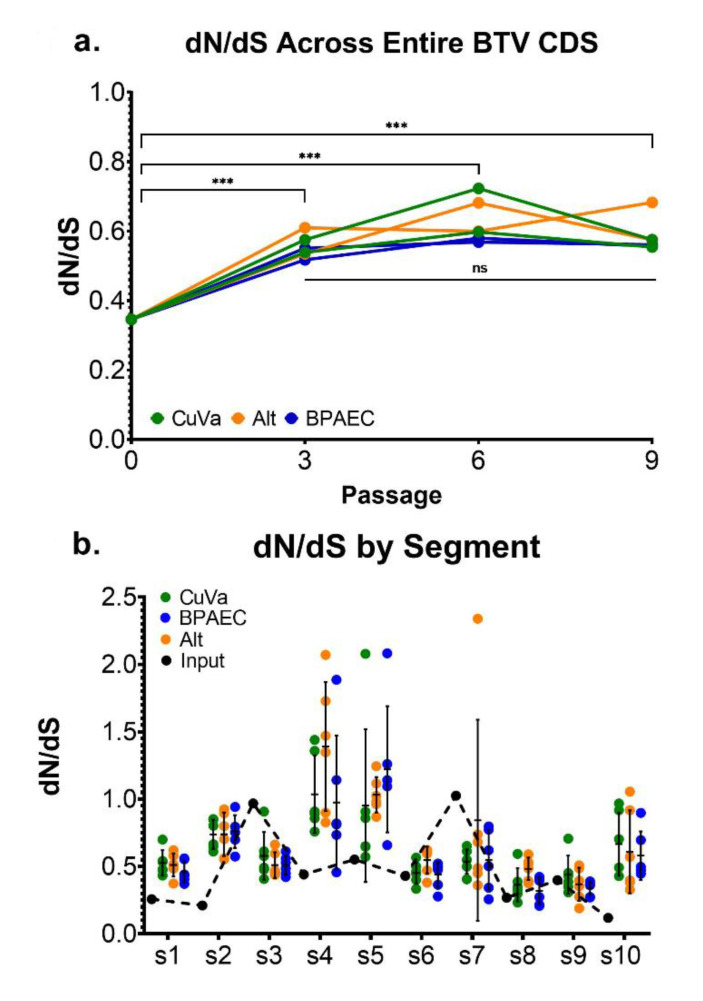
Genetic Selection by Segment and Cell Culture Condition. The proportion of nonsynonymous (dN) to synonymous (dS) changes was used as an estimate of selection. (**a**) dN/dS for each sample was calculated across the entire BTV coding sequence (CDS; inclusive of all ten segments), *** = *p* < 0.0005. (**b**) dN/dS from all passages and replicates are shown. Error bars depict mean and standard deviation of each segment according to cell culture condition. BTV17-INPUT is shown by black dots and the dashed line. Segments 1–10 are represented along the *x*-axis (s1, s2, s3, s4, s5, s6, s7, s8, s9, and s10).

The proportion of nonsynonymous sites across the entire BTV coding sequence (*pN*) for BTV17-INPUT was 0.77, and this measure remained relatively unchanged across segments and samples for subsequent passages. Propagation on BHK cells (BTV17-INPUT) appeared to result in purifying selection for most segments (mean $d_{N}/d_{S}$ = 0.35) (Figure 7a), although segments 3 and 7 had $d_{N}/d_{S}$ ratios closer to 1 (0.97 and 1.03, respectively), indicating more neutral selection (Figure 7b). In contrast, after passage in CuVaW3 and BPAEC cells, segments 4 and 5 generally exhibited positive selection (mean $d_{N}/d_{S}$ across all conditions: 1.13, range 0.60–1.71; and 1.07, range 0.61–1.58, respectively) (Figure 7b). When assessed across the coding sequence of all ten segments, BTV demonstrated negative, or purifying selection, in all conditions and passages. However, purifying selection appeared to be relatively stronger in BTV17-INPUT (*p* < 0.0005, propagated in BHK cells) compared to BTV propagated in BPAEC and CuVaW3 cells. When individual segments were analyzed across passages, $d_{N}/d_{S}$ was varied from passage to passage, and between cell culture conditions (Figure S4).

## 4. Discussion

By using a relaxed system of propagation, we sought to remove the impact of varying host-derived features (i.e., adaptive immunity, overall health status, species variation) on BTV’s genetic diversification, instead capturing the virus’s inherent capacity to diversify in vertebrate and invertebrate cells.

Several studies have demonstrated that arboviruses diversify to a greater extent in the insect host, possibly due to certain invertebrate immune response mechanisms (RNA interference, RNAi) [16,50,51]. While there is evidence for the existence of RNAi in *Culicoides* and *Culicoides*-derived cell lines, the presence of RNAi in the cell line used in our study (CuVaW3) has yet to be demonstrated [52,53].

Contrary to our expectation of increased genetic variation in virus passaged solely in CuVaW3 cells, we found that the number of single nucleotide variants (SNVs) detected at each passage was quite variable. While the consistent detection of SNVs across all conditions likely indicates the presence of population heterogeneity and possible viral quasispecies, overall measures of BTV-17 population diversity remained largely constant regardless of cell type. In contrast, Caporale et al. and Lean et al. found that bluetongue virus passaged in a different *Culicoides*-derived cell line (KC cells) had relatively greater numbers of variants than that of virus passaged in mammalian cells [54,55]. However, our approach with BPAEC cells—a cell and host type that BTV demonstrates a natural tropism for—distinguishes our findings with mammalian cells from those previous studies where embryonated chicken eggs, BSR cells, Vero cells, and CPT-tert cells were used.

Despite passaging BTV-17 in a relaxed model with relatively few constraints on genetic diversification, we found that this virus exhibited marked genetic consistency between passages. Genetic diversity among BTV field isolates most likely reflects numerous factors, including host immune response, vaccination status, host species, infectious titer, possible co-infecting viruses, and bottlenecks that may occur during transmission. While our inability to capture these factors is a limitation of the present work, the underwhelming degree of genetic diversity detected in our study is corroborated by studies demonstrating that the electropherotype of BTV does not change across prolonged infection in ruminant hosts and that experimental in vivo transmission between *C. sonorensis* and sheep and cattle results in minimal changes in the overall genetics of BTV [20,29,30].

Divergence of passaged viruses from the input strain likely indicates that BHK 21 cells exert different selection pressures than BPAEC or CuVaW3 cells. The disproportionately high genetic richness of BTV17-INPUT coupled with unremarkable genetic distance across all segments indicates that most SNVs in BTV17-INPUT are low-frequency. In addition, BTV17-INPUT demonstrated the lowest $d_{N}/d_{S}$ ratio of our samples, indicating relatively dramatic negative selection. These findings may demonstrate that strong purifying selection in BHK cells is coupled with increased frequency of neutral—or even deleterious—alleles that are not purged from the population [56]. This phenomenon, described by Cvijovic et al. using a forward-time model, can result in distortions of genetic measures that mimic population expansion [57]. Alternatively, interferon-deficient BHK 21 cells may promote “tolerance” of viral variants, causing a similar net outcome in our various diversity measures [57,58].

Consistent with the work of Bonneau et al., who found that segment 10 frequently developed nonsynonymous mutations during transmission from *Culicoides* to ruminants, we detected high complexity (Shannon entropy) and divergence (*F_ST_*) for segment 10, regardless of cell culture condition. This suggests that increased population heterogeneity may be a characteristic feature of BTV’s segment 10. While this segment is generally considered to be one of the more conserved BTV segments, some groups have identified relatively high substitution rates for segment 10 [24,59,60]. While underlying mechanisms for this trend are unclear, segment 10 plays an essential role in recruiting RNA segments during viral replication and generation of the cytopathic effect, and thus the low-level genetic heterogeneity detected here and in other studies may be explained by its functional role in virus replication. Alternatively, there may be secondary structures that affect sequencing chemistry and falsely increase the number of variants detected.

Our work corroborates findings by Caporale et al. and Lean et al., who described a decrease in variants in BTV isolated from whole blood, particularly when passaged in BHK 21 cells [54,55]. It is likely that isolating BTV on this non-native cell type causes a population bottleneck and purifying selection. We suspect that the consensus changes that arose in segments 5 and 10 when BTV-17 was transitioned from BHK 21 cells to bovine or *Culicoides* cells may reflect a reversion from BHK-specific adaptations in these segments. While segment 5 and 10 RNAs are known to interact, these interactions occur at different sites than those detected in our work [61]. However, currently uncharacterized RNA–RNA, RNA–protein, and protein–protein interactions in BTV assembly and maturation may exist.

A caveat to this work and all deep-sequencing projects is that variant detection is not free of bias. LoFreq default parameters include stringent quality filters that reduce the incidence of false-positives [40]. However, studies have demonstrated that false positives are relatively common regardless of sequencing platform and variant caller used, and that sensitivity often varies between variant-calling programs [62]. By performing our work in duplicate and eliminating any PCR unnecessary amplification steps, we have tried to reduce as many variables as possible. However, sequencing chemistry, structural features of DNA–DNA interactions, and inherent sequencing errors may all contribute to uncertainty in our data.

In summary, few studies have utilized a comprehensive approach to evaluate contributions of viral genetic diversity and how the existence of multiple genotypes within alternate host passages may influence BTV evolution. Improved understanding of BTV genetic variability during host alternation is critical for predicting the emergence and impact of *Culicoides*-transmitted viruses in different ecosystem contexts with disease transmission models. Additional infection studies in vertebrate and invertebrate hosts, coupled with deep viral sequencing, are warranted to better understand these findings.

## Supplementary Materials

The following are available online at https://www.mdpi.com/1999-4915/12/9/1038/s1, File S1: Modified Schneider’s Drosophila Media. Figure S1: Genetic Distances across Passages and Segments by Cell Culture Condition, Figure S2: Genetic Richness across Passages and Segments by Cell Culture Condition, Figure S3: Genetic Complexity across Passages and Segments by Cell Culture Condition, Figure S4: Genetic Selection across Passages and Segments by Cell Culture Condition, Table S1: Depth of Sequencing Coverage across Samples.
