# Supplementary material for: The Genetic Diversification of a Single Bluetongue Virus Strain Using an In Vitro Model of Alternating-Host Transmission
Source: Viruses. 2020 Sep 18;12(9):1038. doi: 10.3390/v12091038 (PMC7551957; doi:10.3390/v12091038)
Supplement: Supplementary file 1 [file viruses-12-01038-s001.zip › supplementary/supplementary.docx]

Supplemental Materials

**File S1: Modified Schneider’s Drosophila Media.**

Maintenance media for CuVaW3 cells was prepared from a modified recipe based off that described by Weschsler et al., courtesy of recommendations from collaborators at USDA-ARS in Manhattan, KS.^1,2^

Add the following to 1 liter of HyClone cell culture grade water:

24.5 g powdered Schneider’s Drosophila Media

0.4 g sodium bicarbonate

0.06 g L-glutamine

0.006 g reduced glutathione

0.03 g L-asparagine

2.1 g sodium hydroxide pellets

0.6 g calcium chloride

Adjust pH to ~6.7 with 12.1 N HCl

Sterile-filter solution with 0.22 µm vacuum filter

Add 15% heat-inactivated, insect-cell tested fetal bovine serum

Reference

1. Wechsler SJ, McHolland LE, Tabachnick WJ. Cell lines from Culicoides variipennis (Diptera: Ceratopogonidae) support replication of bluetongue virus. *J Invertebr Pathol*. 1989;54:385-393.

2. McHolland LE, Mecham JO. Characterization of cell lines developed from field populations of Culicoides sonorensis (Diptera: Ceratopogonidae). *J Med Entomol*. 2003;40(3):348-351. doi:10.1603/0022-2585-40.3.348.

**Table S1. – Depth of Sequencing Coverage across Samples*.*** The mean depth of sequencing coverage across the coding sequence of each BTV segment of BTV17-INPUT and all samples from passages 3, 6, and 9 are shown.


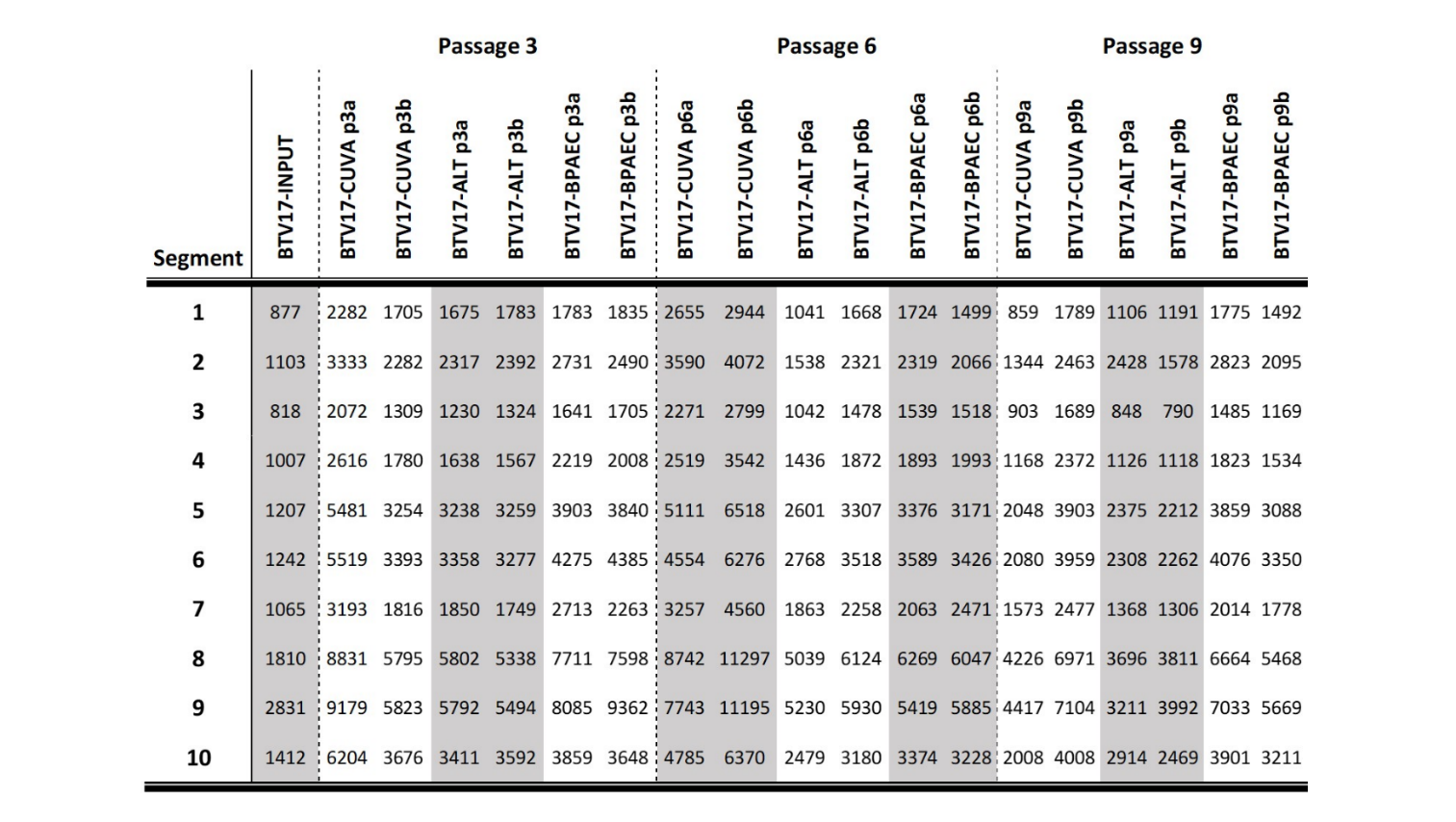


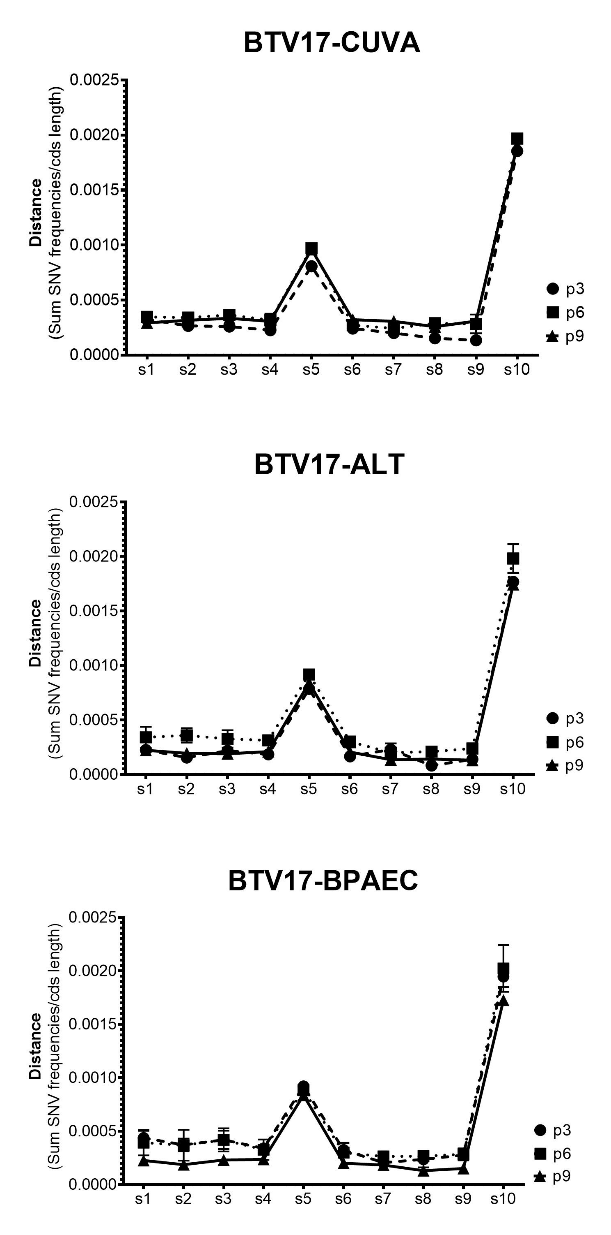


**Figure S1. – Genetic Distances across Passages and Segments by Cell Culture Condition.** Genetic distances (i.e., sum of single nucleotide variant (SNV) frequencies per segment) are normalized by each segments’ coding sequence length. Mean distance (and range) for each segment and passage is shown according to cell culture condition. Virus harvested from passage 3 is connected by dashed line connecting circles; virus from passage 6 is connected by dotted line connecting squares; virus from passage 9 is connected by solid line connecting triangles.


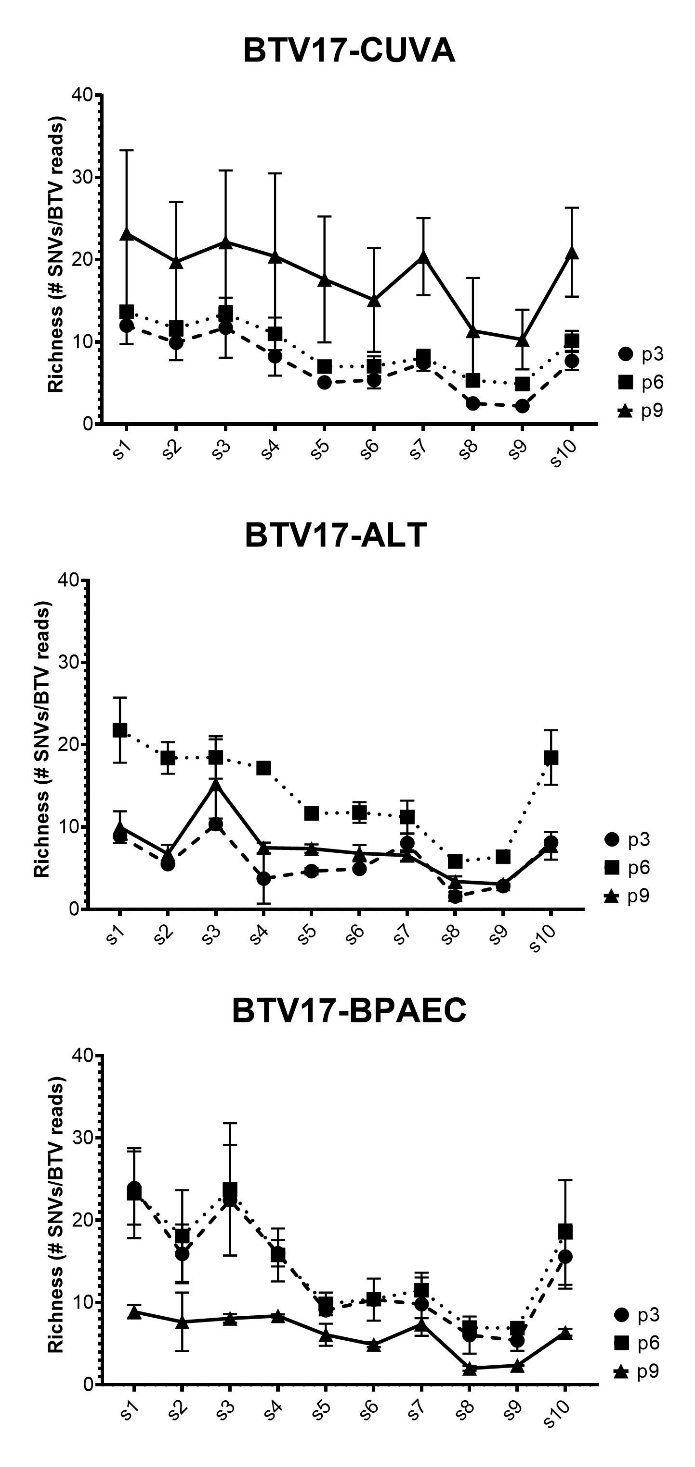


**Figure S2. – Genetic Richness across Passages and Segments by Cell Culture Condition.** Richness of each segment was calculated as the sum of single nucleotide variants (SNV) sites normalized by the number of BTV reads (i.e., variant sites per 10,000 BTV reads). Mean richness (and range) for each segment and passage is shown according to cell culture condition. Virus harvested from passage 3 is connected by dashed line connecting circles; virus from passage 6 is connected by dotted line connecting squares; virus from passage 9 is connected by solid line connecting triangles.


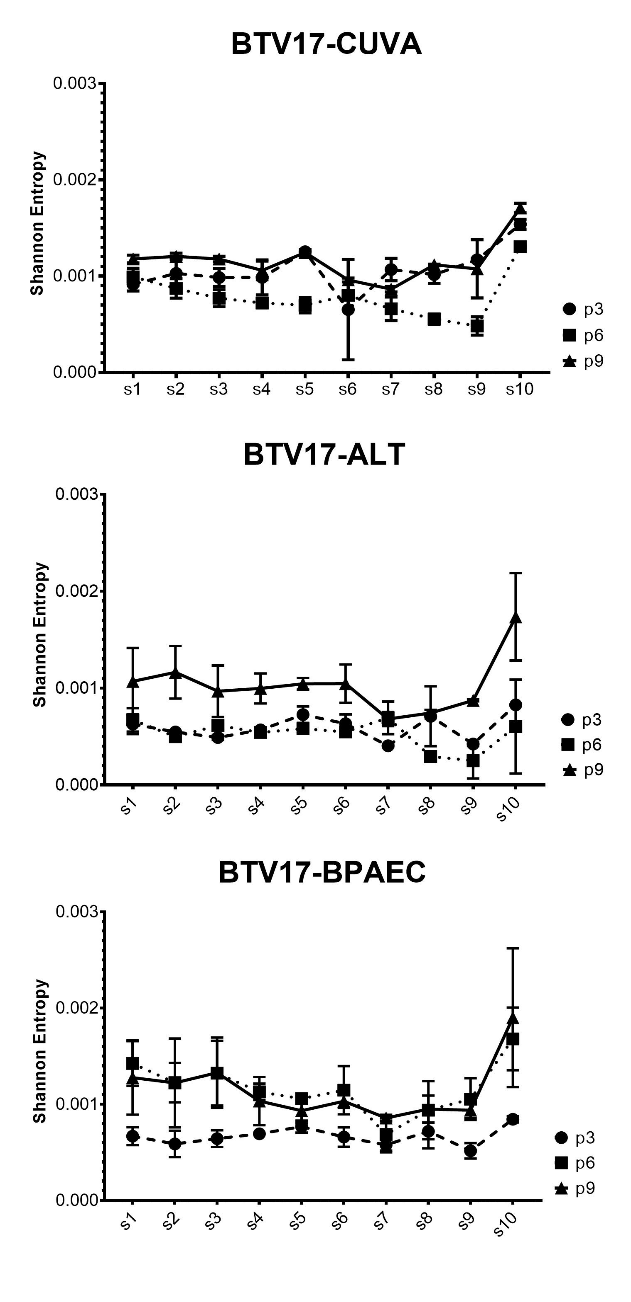


**Figure S3. – Genetic Complexity across Passages and Segments by Cell Culture Condition.** Shannon entropy was calculated as a measure of population complexity across viral coding sequences. Mean Shannon entropy (and range) for each segment and passage is shown according to cell culture condition. Virus harvested from passage 3 is connected by dashed line connecting circles; virus from passage 6 is connected by dotted line connecting squares; virus from passage 9 is connected by solid line connecting triangles.


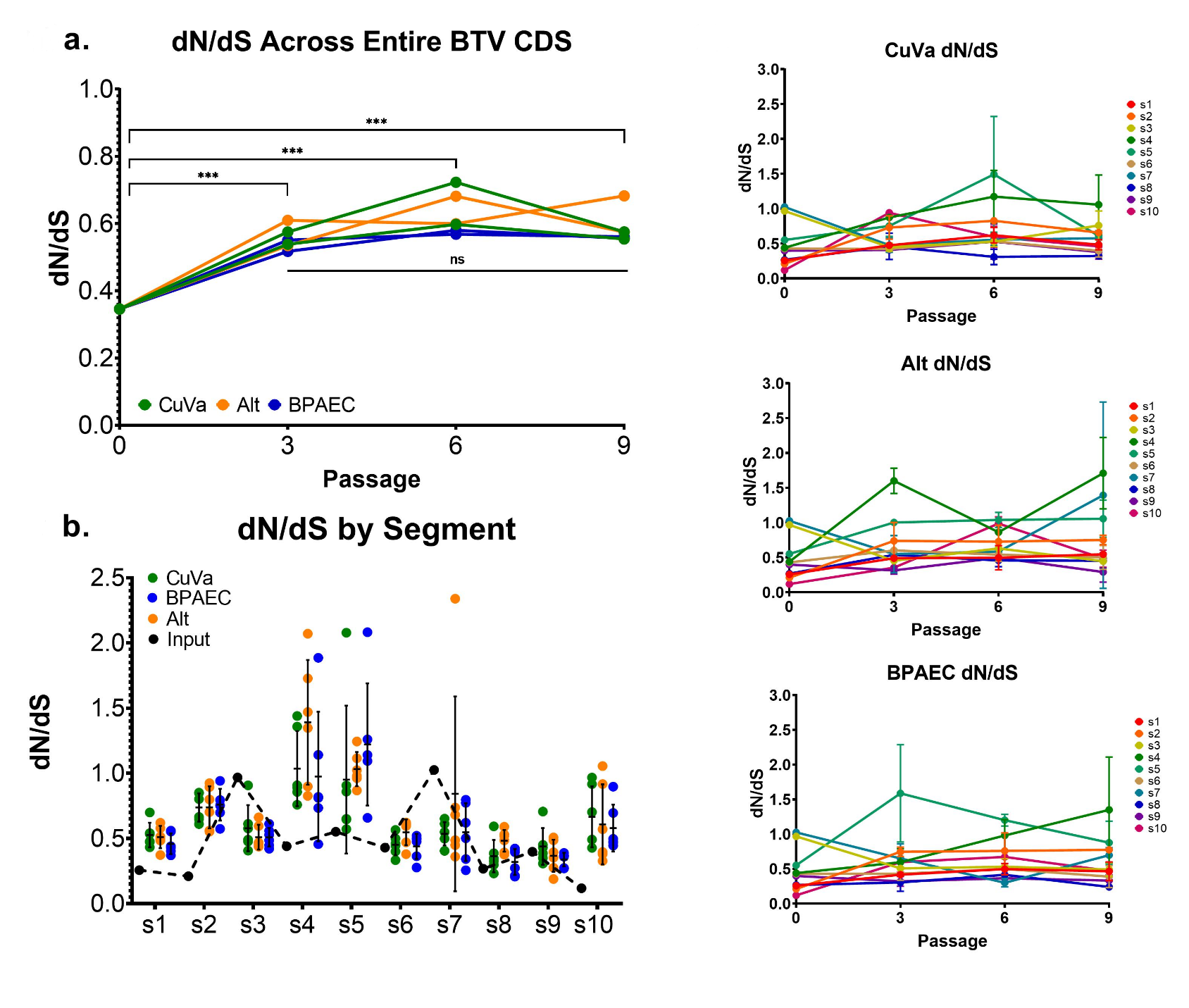


**Figure S4. Genetic Selection across Passages and Segments by Cell Culture Condition.**  The proportion of nonsynonymous (dN) to synonymous (dS) changes was used as an estimate of selection. dN/dS of each segment is shown by passage and each cell culture condition. Error bars represent mean and range for each segment. dN/dS for each segment at passages 0, 3, 6, and 9 are shown. Segments 1-10 are represented by different colored lines (s1, s2, s3, s4, s5, s6, s7, s8, s9, and s10).
